# Synthesizing the latest guideline-based recommendations for the management of female hypogonadism

**DOI:** 10.20945/2359-4292-2025-0395

**Published:** 2025-11-24

**Authors:** Bruna Barbar, Wessam Osman, Channa N. Jayasena, Richard Quinton

**Affiliations:** 1 Hospital de Base, Faculdade de Medicina de São José do Rio Preto, São José do Rio Preto, SP, Brasil; 2 National Diabetes and Endocrine Centre, Royal Hospital, Muscat, Oman; 3 Department of Metabolism, Digestion & Reproduction, Imperial College London, London, UK; 4 Section of Investigative Medicine, Hammersmith Hospital, Imperial College London, London, UK; 5 Northern Region Gender Dysphoria Service, Cumbria, Northumberland, Tyne & Wear NHS Foundation Trust, Newcastle-on-Tyne, UK

**Keywords:** Female hypogonadism, premature ovarian insufficiency, hypothalamic amenorrhea, Turner syndrome, hormone p>replacement therapy, estradiol

## Abstract

Over the past year, three new key guidelines have been published in the area of female hypogonadism, one from the Society for Endocrinology covering the full spectrum of causes of female hypogonadism in adult life, which will form the core of this review; another solely covering premature ovarian insufficiency from a consortium comprising the International Menopause Society (IMS), the European Society of Human Reproduction & Embryology (ESHRE) and the American Society for Reproductive Medicine (ASRM) that updates the 2016 ESHRE guidance, and a third covering Turner syndrome across all stages of life from the International Turner Syndrome Consensus Group. In this review, we aim to synthesize the key elements from all of these documents, providing a timely update for clinicians managing affected women.

## INTRODUCTION

Over the past year, three new key sets of guidelines have been published in the area of female hypogonadism (FH), comprising Society for Endocrinology (SfE) guidance covering the full spectrum of causes in affecting women of reproductive age, which will form the core of this review (^1^); guidance specifically for POI from an international consortium comprising the International Menopause Society (IMS), the European Society of Human Reproduction & Embryology (ESHRE) and the American Society for Reproductive Medicine (ASRM) that updates the 2016 ESHRE guidance (^2^), and for Turner syndrome across all stages of life from the International Turner Syndrome Consensus Group (^3^). In this overview of FH, we aim to review the evidence, with an emphasis on highlighting novel or important new concepts arising from these latest guidelines.

FH is defined as the lack of ovarian reproductive hormone secretion during the normal post-menarcheal to premenopausal age range and is usually associated with prolonged amenorrhea and subfertility (^1^). It is classified into primary and secondary forms, and this distinction has important implications. Primary hypogonadism usually reflects an intrinsic depletion of hormone-secreting follicles in the ovaries, and principally results from gonadal dysgenesis, oophorectomy, or premature ovarian insufficiency (POI), but can also be due to rare disorders of estradiol synthesis or the secretion of biologically inactive gonadotropins.

As with normal menopause, it is associated with abnormally high levels of follicle-stimulating hormone (FSH: >25 IU/L) and luteinizing hormone (LH) due to the loss of central negative feedback by ovarian steroid and peptide hormones. Secondary (*i.e.*, hypogonadotropic or central) hypogonadism results from ovarian under-stimulation due to inadequate gonadotrophin secretion, which translates into low (or inappropriately normal) FSH and LH levels with low estradiol and/or a thin endometrium.

FH presents with a multitude of symptoms, including prolonged amenorrhea, diminished physical, mental and emotional well-being, subfertility and impaired muscle, bone, urogenital and sexual health. Vasomotor symptoms commonly occur in adult-onset FH and are universal with acute surgical or medical oophorectomy in premenopause, but are rare in FH of congenital origin or of prepubertal onset; in the latter, there is instead a failure to fully develop external and internal secondary sexual characteristics or to experience normal menarche. Amenorrhea is classified as primary (absence of menarche by age 15) or secondary (lack of menses for 3 months if previously regular cycle, or for 6 months if cycle was previously irregular). Whether amenorrhea was primary or secondary maps approximately onto whether FH was of prepubertal or postpubertal onset.

## APPROACH TO THE DIAGNOSIS AND CLASSIFICATION OF FH

It is vital to measure gonadotropin levels in order to distinguish primary from central hypogonadism, because the approach to the second-order investigations and fertility counselling is notably divergent (e.g., karyotype or copy number variation in primary FH, and serum prolactin and MRI pituitary in secondary FH). Other investigations are required for determining etiological causes within these two categories and may be ordered in case-by-case settings depending on the associated clinical manifestations present. Indeed, certain non-reproductive defects allow a fairly confident presumptive diagnosis of FH to be made even in normal prepubertal childhood, such as anosmia (suggesting Kallmann syndrome), or the constellation of clinical features that characterize Turner syndrome. In these individuals, pubertal induction with estradiol should be anticipatory from age 11 years, rather than reactive to the experience of pubertal delay (^4^).

Primary FH is always caused by organic pathology. However, secondary FH may be either organic (e.g., due to a genetic defect of hypothalamic-pituitary hormone secretion, parasellar mass lesion, radiotherapy, traumatic brain injury or surgery), or functional/reversible due to non-gonadal illness (e.g., systemic illness, low fat mass, excessive exercise or stress), opiate intake/treatment, or hyperprolactinaemic states. In functional secondary FH (commonly known as hypothalamic amenorrhea - HA), there are two key clinical judgments to make, one diagnostic and the other therapeutic. First, a judgement needs to be made as to how exhaustively to exclude organic disease by, for instance, checking anterior pituitary function, iron studies and/or MRI scanning. Second, for how long should one persist in attempting to achieve remission by targeting the underlying cause, e.g., through behavioural treatments, versus prescribing hormone replacement therapy (HRT)?

Although diagnosing primary FH or POI should be straightforward, patients commonly experience prolonged delays in accessing appropriate treatment; the diagnosis took longer than two years in 23% of Australian women, with at least two clinicians having been consulted on average (^5^) and, in a low-income (Medicaid) urban USA population, the median time from first presentation to diagnosis was 4 years (^6^). These delays may be related to clinicians finding it difficult to even consider the possibility of menopause in younger women, in situations when there are other potential causes for amenorrhea, or in the early years after disease onset when amenorrhea is not sustained and there may be intervals of cyclical bleeding. The latest international POI guidance has therefore commendably attempted to streamline the diagnostic process by no longer requiring a second raised FSH (>25 IU/L) concentration for diagnostic purposes (^2^).

The diagnosis of FH is even more difficult for secondary causes, where the diagnostic guiderail of a raised FSH concentration (>25 IU/L) is lacking and clinicians may not readily consider checking the estradiol concentration. Even if available they may be uncertain as to how to interpret the result. Specifically, laboratory printouts giving normative ranges for serum estradiol across the female menstrual cycle are unhelpful because healthy menstruating women rarely need measurements of estradiol levels. The range of quoted values (typically 1002,000 pmol/L) is irrelevant to the context of prolonged amenorrhea where any temporal relationship to the menstrual phase is necessarily lacking.

Therefore, based on the range of estradiol concentrations observed in studies of women with prolonged lactational or hypothalamic amenorrhea (HA), SfE guidance recommends that a serum estradiol concentration < 200 pmol/L associated with prolonged amenorrhea is suggestive of FH (^1^).

The importance of early diagnosis and treatment of FH cannot be overstated. Aside from issues of patient symptoms and quality of life, prolonged hypoestrogenism is an important risk factor for cardiovascular disease, osteoporosis and fracture, and possibly even dementia (^7^,^8^). Although estrogen replacement therapy in preventing dementia remains controversial in the literature (^9^), there are established benefits to both cardiovascular and bone health in women with POI (onset prior to age 40 years) and early menopause (onset 40-45 years) (^1^,^10^). Considering that most hormone replacement therapy (HRT) studies have been conducted with post-menopausal women without specifying a menopausal age onset, further research is still needed in younger populations and, lacking large clinical trials, patient registries are likely to provide key data.

## THE REGULATION AND PHYSIOLOGICAL ROLES OF OVARIAN HORMONES

Gonadotropin-releasing hormone (GnRH)-stimulated FSH and LH pulses of increasing amplitude from the pituitary gland herald the onset of ovarian steroidogenesis, corresponding to thelarche (Tanner B2). Estradiol secreted by the granulosa cells of developing follicles in response to FSH is central to the acquisition of key secondary sex characteristics and growth and maturation of the uterus over a period of 3-4 years. This highlights the importance of an appropriate tempo of incremental estradiol administration so as to mimic normal puberty, avoiding premature progesterone exposure, which can limit full development of the breast by limiting the branching morphogenesis necessary for complete development of the ductal tree (^4^,^11^), and may also limit uterine development (^12^). Although pulsatile subcutaneous GnRH treatment has been used both to induce puberty in girls and, more commonly, to achieve ovulation in adult females with central hypogonadism of hypothalamic origin, its complexity, the associated costs and availability in only very few countries, combine to explain why it is rarely used nowadays (^13^).

GnRH neurons are not a site of any significant estradiol negative feedback, and the secretion of GnRH at their terminals at the median eminence is directly regulated by Kisspeptin (Kp) secreted by neurons having their cell bodies in the arcuate (ARC) and anterior periventricular (APV) nuclei of the hypothalamus. Kp secretion is determined through the integration of both external environmental and internal homeostatic inputs, including leptin secreted by fat cells and estradiol, progesterone and anti-Müllerian hormone (AMH) secreted by the ovaries. Broadly, estradiol inhibits Kp secretion by KNDy neurons in the ARC, but stimulates Kp secretion by APV neurons, thereby forming the oscillator circuit that creates cyclical hormone secretion and gametogenesis in females compared to the steady-state system in males (^14^). Other peptides secreted by KNDy neurons that help to regulate GnRH secretion include neurokinin B (NKB - stimulatory) and dynorphin (inhibitory). Crucially, inactivating mutations of Kp, NKB and GnRH peptide hormones, or far more common of their G-proteincoupled receptors, are among the recognized genetic causes of congenital hypogonadotropic hypogonadism (CHH) (^15^). In contrast, the higher concentrations of AMH (secreted by granulosa cells in small antral and preantral ovarian follicles) promote a higher frequency of GnRH pulses that favors LH secretion, as occurs in PCOS.

In reproductive life, estradiol is essential for cyclical endometrial repair and cellular proliferation after each menstruation during the follicular phase, and for the expression of endometrial progesterone receptors, thereby underpinning the necessary changes that are required for embryo implantation and the establishment of pregnancy (^16^). It is also essential for the proliferation of epithelial mucosal cells of the vagina and vulva, maintaining the integrity and lubrication of the vulvo-vaginal epithelium, and for the ongoing health and functionality of the urogenital tract.

Estradiol promotes epiphyseal fusion and is also crucial to achieving peak bone mass and maintaining it thereafter. During puberty, the rise in sex hormone levels, particularly estradiol, stimulates osteoprotegerin activity and decreases RANKL expression in osteoblasts. Estradiol also inhibits the secretion of proinflammatory cytokines, which in turn reduces osteoclastic activity, leading to decreased bone resorption and increased repair of microfractures, thereby augmenting trabecular bone thickness in particular. In contrast, estrogen deficiency promotes osteoclastic activity and subsequent bone resorption via several pathways, including tumor necrosis factor-α, interleukin-1β, RANK-ligand and sclerostin (Wnt signaling) pathways (^1^), which may also be exacerbated by upstream disturbances in extragonadal reproductive hormones (^17^).

Estradiol plays an important role in modulating cardiovascular risk through its signaling via ER-alpha and ER-beta receptors. As oxidative stress plays a significant part in the pathogenesis of atherosclerosis, myocardial dysfunction, cardiac hypertrophy, heart failure, and myocardial ischemia, excess reactive oxygen species (ROS) resulting from hypoestrogenic states lead to the increased 10-year cardiovascular risk observed on POI and early menopause. Estradiol is thought to act in the upregulation of antioxidant gene expression, increasing Endothelial nitric oxide synthase (eNOS) activity and decreasing superoxide production, with subsequent reduced apoptosis and necrosis of cardiac and endothelial cells. Altogether, the ensuing actions result in a more favorable lipid profile with increased levels of HDL and decreased levels of LDL and total cholesterol (^18^,^19^). Estradiol also promotes cerebral blood flow and vasodilation and reduces oxidative stress and neuroinflammation, as well as enhancing functioning of the hippocampal and prefrontal cortices (^20^,^21^). Additional effects of estradiol on mitochondrial activity, regulation of insulin sensitivity and the renin-angiotensin-aldosterone system further contribute to its broadly favorable metabolic effects (^22^,^23^).

## CAUSES OF FH

### Primary hypogonadism, including premature ovarian insufficiency (POI)

POI is characterized by oligo-amenorrhea (>4 months duration) in conjunction with a raised FSH level (>25 IU/L), occurring in women <40 years old and having characteristic signs or symptoms, including sleep disturbance, vasomotor fluctuations, low libido and energy, altered urinary frequency, dyspareunia due to vulvovaginal atrophy and cognitive disturbances. Recent international POI guidelines indicate a prevalence of 3.5% and recommend that a single FSH assessment (>25 IU/L) in a symptomatic woman is sufficient for diagnosis; a second FSH measurement four weeks later only being required where there is diagnostic uncertainty (^2^).

Although the etiology of most cases of POI remains undefined based on present knowledge and techniques, further investigation of the cause should nevertheless be undertaken, including a personal and family medical history, karyotype (or copy number variation test), thyroid-stimulating hormone (TSH) level, thyroid peroxidase (TPO) antibody concentration, and fragile-X testing (*FMR1* gene). Genomic evaluation should be performed in younger women once other potential causes have been excluded, but from the perspective of other family members, there are benefits in making it across the age spectrum of POI, especially in respect of *FMR1* (^2^).

Women with primary amenorrhea and absent puberty are likely to have gonadal dysgenesis (see below), although type 1 galactosemia (*GALK1* gene), *FMR1* gene mutations and even Turner syndrome (TS) can equally present with secondary amenorrhea post-puberty. Autoimmune oophoritis is a major postpubertal cause, albeit anti-ovarian antibody tests are non-specific, and so the diagnosis is usually inferred from the presence of other autoimmune conditions, thyroid, adrenal, celiac disease, or pernicious anemia.

#### Gonadal dysgenesis and Turner syndrome

Gonadal dysgenesis refers to a broad range of conditions characterised by impaired gonadal development due to alterations of genetic material or abnormalities in cell division that lead to dysplastic or streak gonads and a prepubertal female phenotype irrespective of sex chromosomes. The karyotype can thus be 46XX, 46XY (Swyer syndrome), or 45X0 (or variants thereof) in Turner syndrome (TS). Unless there were other characteristic features leading to the clinical suspicion and diagnosis of TS in prepuberty, gonadal dysgenesis otherwise presents invariably with primary amenorrhea and absent puberty.

TS is the most prevalent sex chromosome disorder in women, affecting 5 per 10,000 live births (^24^). There is complete or partial absence of one X chromosome, with ovarian insufficiency primarily associated with deletions or structural abnormalities of the long arm (Xq13-27), which contains genes critical for ovarian development and function, and short stature with haplo-insufficiency for the *SHOX* gene on the pseudo autosomal region 1 of the short arm (Xp22.3). In early fetal life, there is accelerated oocyte apoptosis and impaired formation of primordial follicles, leading to depletion of germ cells (^25^). Characteristic intrauterine features on prenatal sonography comprise increased nuchal translucency, cystic hygroma, intrauterine growth restriction and cardiac anomalies, but a minority of cases entirely lack any notable physical dysmorphisms, whether *in utero* or postnatal life. Longitudinal studies have identified abnormally raised FSH and LH concentrations during postnatal minipuberty and from late prepuberty onwards.

If the diagnosis of TS was made in prepuberty, then annual measurement of FSH, LH and AMH concentrations should be undertaken from age 8-9 until 11-12 years, to identify those girls who will need early pubertal-induction proactively. AMH levels < 4 pmol/L and raised FSH are highly correlated with absent puberty (^3^) and, in these girls, estrogen replacement should not be delayed until thelarche fails to appear at the upper limit of the normal age of onset; rather it should begin at 11 years of age with incremental doses of 17β-estradiol until the adult replacement dosage is reached over the next 3-4 years, and with longitudinal monitoring of Tanner staging and sonographic assessment of uterine dimensions and maturity (^2^-^4^). It is also recommended that the final adult dose should achieve serum estradiol concentrations in the 350-550 pmol/L (100-150 pg/mL) range as this seems to be associated with better uterine maturation (^3^). Nevertheless, POI guidance still does not recommend adjusting HRT doses according to the serum estradiol concentration (^2^).

The TS phenotype is highly variable, depending to some extent on whether all or part of the X-chromosome is missing and whether there is mosaicism (albeit the ratio of total somatic cells with normal or abnormal chromosomes cannot be reliably extrapolated from that observed in culture of peripheral blood leukocytes). The initial presentation and diagnosis may thus arise as a result of prenatal diagnostics, childhood growth retardation, failure of puberty with primary amenorrhea, or even with the onset of secondary amenorrhea due to POI (^25^). Up to one third of girls with TS may develop spontaneous thelarche, but spontaneous menarche only occurs in 5%-20% and < 10% go on to develop regular periods, albeit with an increased risk of miscarriage in pregnancy and an unquantified increased risk of developing POI in later life.

Natural conception is not possible for women with gonadal dysgenesis and streak ovaries, in whom eggdonation is the only means of carrying a pregnancy to term, but pregnancy may still occur in up to 10% of TS women, being the vast majority of cases among those with mosaic karyotypes and a history of spontaneous menarche. For TS women of reproductive age who are menstruating normally and have the necessary psychological maturity (but are either not in a relationship or not ready to start a family), oocyte cryopreservation is recommended. Contraceptive measures should also be considered in those who are sexually active if pregnancy is not desired. Although a potentially promising technique for younger TS girls (^26^), ovarian tissue preservation should only be offered in the context of a formal research protocol (^3^).

#### Autoimmune

Autoimmune oophoritis accounts for up to 30% of cases of POI, most commonly associated with thyroid and adrenal disturbances, but less frequently pernicious anemia, T1 diabetes mellitus, rheumatoid arthritis, Crohn’s disease, myasthenia gravis and lupus (^27^). Up to 60% of cases diagnosed with APS-1 (autoimmune polyendocrinopathy-candidiasis-ectodermal dystrophy - APECED), characterized by mutations in the autoimmune regulator (*AIRE*) gene, will develop POI. There is a significant correlation between positive adrenal 21-hydroxylase antibodies and POI, and monitoring of adrenal as well as thyroid function should be considered in younger females with POI of unknown cause, albeit POI usually presents a decade before the onset of clinical adrenal involvement (^27^).

Women may experience brief disease remissions that allow them to ovulate and fall pregnant, particularly in the early years after onset when AMH levels are > 4 pmol/L, and with an approximately 5% lifetime chance of conceiving naturally (^28^). Although nearly three-quarters of women with 46XX POI retain ovarian follicles, chronically raised LH levels promote premature luteinization that diminishes the chances for spontaneous ovulation over time (^1^). Despite promising results in murine models (^24^), there are no disease-modifying therapies for autoimmune oophoritis, albeit the impact of oophoritis caused by cytotoxic drugs such as cyclophosphamide, can be mitigated by contemporaneous treatment with a GnRH-analogue to induce gonadotropin suppression and ovarian quiescence (^29^).

#### Iatrogenic

In addition to the obvious immediate implication of bilateral oophorectomy, removal of benign ovarian cysts (especially if large or the surgery is repeated) predisposes to POI. Moreover, there is also a growing population of young women who have survived cancer and its treatment. The risks from chemotherapy and pelvic radiotherapy vary by regimen, dose, and age at administration. The high toxicity is associated with alkylating agents such as busulfan and cyclophosphamide, and high-dose lomustine; larger doses and older age are linked to poorer outcomes in terms of ovarian function (^30^-^32^). If time permits, patients should always be referred for a consultation regarding strategies for fertility preservation prior to undergoing treatment (^32^). Many younger patients later recover gonadal function following an initial phase of chemotherapyinduced POI, and a higher AMH level is predictive of this (^33^), but their depleted ovarian reserve may later predispose them to POI or early menopause (^34^,^35^).

Finally, even if pregnancy is achieved, previous pelvic radiotherapy limits the ability of the uterus to support pregnancy to term, resulting in a much higher likelihood of miscarriage, preterm labor, low birth weight and other complications (^36^).

#### Genetic mutations and environmental factors

A premutation in *FMR1* is the most common single gene abnormality associated with the development of POI, accounting for 3%-5% of cases; some 12.9%24% of women carrying it will experience POI, with a younger age of onset occurring across successive generations (“anticipation”) (^37^) and has been linked to an increased risk of mental retardation, tremor and ataxia syndrome in affected males (^38^). However, more extensive genetic screening is now becoming a standard of care in POI, with one study suggesting a prevalence of genetic defects in up to 23% of younger patients and those presenting with primary amenorrhea (^39^). Nevertheless, data from the UK Biobank suggest that, for the vast majority of women, POI is not commonly caused by autosomal dominant variants of genes previously reported or currently evaluated in clinical diagnostic panels; most cases instead likely being oligogenic or polygenic in nature (^40^). However, further studies in different populations are still needed to solidify recommendations and to better understand the roles of gene regulatory pathways and transcription factors in ovarian development and follicle maturation.

Environmental factors, such as cigarette smoking, hysterectomy, recurrent viral infections, exposure to phthalates, bisphenol A and pesticides, as well as Endocrine Disrupting Chemicals (EDCs), have also been linked to an earlier onset of menopause, most likely via an epigenetic effect or, potentially, an impact of on the ovarian vascular supply depending on the individual risk factor (^41^).

### Central hypogonadism

Central hypogonadism in women results in impaired ovarian function due to lack of gonadotropin stimulation. It may be congenital or acquired and, if acquired, either organic or functional. Congenital forms are among the very rare causes of FH, including CHH (female prevalence of 1-in-40 to 125,000), CHARGE syndrome (see below; 1-in-15,000), combined pituitary hormone deficiency (CPHD; 1-in-5 to 10,000) and septo-optic dysplasia (SOD; 1-in-10,000), with perhaps 60% of the latter two exhibiting gonadotropin deficiency.

In contrast, hypothalamic amenorrhea (HA), a form of functional central FH, is among the most frequent causes of amenorrhea during reproductive life, and is by far the most common cause of FH. Other frequent causes of functional central FH include hyperprolactinemia, whether induced by prolactinoma, with serotoninergic and anti-dopaminergic drugs, opiates, or depot injections of medroxyprogesterone acetate (MPA). It is likely that, in many cases, functional CH may result from a combination of predisposing factors that individually might not be sufficiently severe to disturb menstrual cyclicity.

Nevertheless, similar principles of diagnosis and treatment apply to all forms of CH (^42^) and, indeed, there are shared genetic predispositions in relation to CHH, CPHD, SOD, CHARGE and HA (^43^-^46^).

#### Congenital hypogonadotropic hypogonadism (CHH)

This is characterized by a congenital defect of GnRH secretion or (less commonly) GnRH action. Around two-thirds of CHH patients present with absent puberty, which is associated with complete GnRH/ LH apulsatility, and the remaining one-third exhibit arrested partial puberty at presentation, which is associated with low-frequency, low-amplitude, or nocturnal-only pulse patterns. However, primary amenorrhea is ubiquitous in any case. In a European web-based survey, the median age at diagnosis and clinically meaningful treatment in females was 20.7 ± 7.4 years by patient recall, probably due to diagnostic confusion by their clinicians with self-limiting delayed puberty (SLDP) or HA, both of which share a common biochemical signature with CHH. This delay in diagnosis and effective treatment was associated with enduring psychological and psychosexual morbidity (^47^).

CHH can occur as an isolated neuroendocrine defect in 30%-40% of cases or in association with other developmental anomalies, most commonly anosmia that defines Kallmann syndrome (around 50% of cases), but also hearing loss (10%), midline defects (cleft lip/palate - 5%), synkinesia, renal agenesis, dental and skeletal anomalies (collectively around 5%). The link between anosmia (lack of sense of smell) and GnRH deficiency is explained by the extracranial origin of GnRH neurons within the embryonic olfactory placode and their migration alongside fascicles of the olfactory, terminal and vomeronasal nerves during fetal life (^42^,^48^,^49^).

Pathogenic mutations in over 60 genes have been linked with CHH, acting either alone or in combination (oligogenic disease), with perhaps 50% of cases now having a robust genetic explanation. Mutations that disrupt GnRH neuron development and migration usually manifest as Kallmann syndrome (KS), whereas those that disrupt GnRH homeostasis, secretion or action present exclusively as pure neuroendocrine CHH. Synkinesia and renal agenesis are particularly associated with *ANOS1* mutations, hearing loss with *CHD7* and *SOX10* mutations, and midline, digital and dental defects with mutations of *FGFR1* or its cognate ligand *FGF8*. Given that X-linked KS (*ANOS1*) comprises only around 10% of KS cases (5% of total CHH), the genetic architecture of CHH provides no clues as why it is around 3-4-times less common in females (^15^).

#### Hypopituitarism and combined pituitary hormone deficiency (CPHD)

Acquired hypopituitarism in adulthood is most commonly caused by benign parasellar tumors, comprising in descending order of frequency, pituitary adenomas (most commonly prolactinomas or non-functioning), craniopharyngiomas, Rathke’s cleft cysts, meningiomas, gliomas and germinomas. Prolactinomas cause functional CH through both hormonal and mass effects. Other causes include CNS infections, traumatic brain injury (especially military blast trauma), radiation, surgery and, rarely nowadays in the developed world, Sheehan’s syndrome (pituitary infarction caused by severe post-partum hemorrhage) (^50^).

Combined pituitary hormone deficiency (CPHD) is defined by the deficiency of at least two pituitary hormones and is often diagnosed and treated postnatally or in early childhood. However, gonadotrophin deficiency may not become apparent until puberty fails to initiate or, as hormone deficiencies may manifest metachronously, with the onset of secondary amenorrhea in later life. Several genes have been identified, comprising either pituitary transcription factors or CHH-related genes, but the vast majority of cases remain without an identified genetic cause (^51^-^53^). There are characteristic MRI appearances of pituitary fossa hypoplasia and/or posterior pituitary ectopia that are also seen in isolated GH deficiency. SOD is a related developmental brain malformation that can present with pituitary hormone deficiencies, severe visual impairment, neurocognitive disability and neurodivergent traits, as well as agenesis of the corpus callosum or optic nerve hypoplasia (^54^).

#### CHARGE syndrome

The constellation of coloboma (ocular malformation of the lens, iris, or retina), congenital heart defects, choanal atresia (abnormal formation of the nasal cavity), retardation of growth and development, genital hypoplasia, and ear anomalies (both external and internal) associated with deafness define CHARGE syndrome, a condition of multiple congenital anomalies that also frequently includes severe learning difficulty, facial asymmetry and immunologic problems (^55^). Most patients exhibit CHH, with or without anosmia, and it may not be easy to distinguish between milder forms of CHARGE syndrome from the more severe KS phenotypes. Approximately two-thirds of cases are explained by large *de novo* deletions in the Chromodomain-helicase-DNA-binding protein 7 (*CHD7*) and, although the same gene is also involved in both normosmic CHH and KS, inherited point mutations are more typical in these conditions (^56^).

#### Prader-Willi syndrome

Prader-Willi syndrome (PWS) is a rare genetic disorder (1/10,000-25,000), associated with severe hypothalamic dysfunction caused by lack of expression of the paternal copy of maternally imprinted genes in the chromosome region 15q11-13. Subtypes are classified into deletions (70%), maternal uniparental disomy (25%-30%), imprinting center defects (3%5%) and rare unbalanced translocations. It typically causes profound physical, mental and social disability. Puberty is usually delayed or incomplete due to CHH, but may occasionally be precocious, and females are usually infertile. As well as HRT, patients typically benefit from growth hormone treatment during childhood and adolescence and, crucially, long-term management of hyperphagia and obesity (^57^).

#### Functional central FH including hypothalamic amenorrhea

##### Definition

Functional central FH is fully reversible when the external constraint to gonadotropin (or GnRH) secretion is removed. One form of this, hypothalamic amenorrhea (HA), is among the most common pathological causes of secondary amenorrhea, and results from suppression of GnRH secretion by an active systemic disease process, stress, or a state of relative energy deficit, whether from excessive exercise or disordered/restricted food intake (^58^). It is likely to have been programmed by evolution to prevent pregnancy during times of scarcity or long-distance migration. Although most women and girls presenting with HA do not have an active eating disorder such as anorexia nervosa, many will have disordered eating or a history of eating disorder, along with high-achieving or perfectionist personality traits and sleep deprivation.

##### Pathophysiological basis and differential diagnosis of HA

Secretion of leptin by adipose cells is heavily influenced by energy balance and fat mass, and leptin deficiency inhibits Kp secretion. Reduced Kp secretion initially slows GnRH pulse frequency, which favors FSH secretion as per early puberty, but with extremely low leptin levels signaling critical undernutrition, the reproductive axis then shuts down entirely. Nevertheless, whereas infusions of pulsatile GnRH and twice daily subcutaneous injections of leptin fully restored reproductive axis function in women with HA (^59^), the effect of Kp infusions was less marked and not sustained (^60^).

South Asians appear more protected from HA due to higher fat mass than Caucasians with a similar BMI, whereas athletes may exhibit HA with normal (or even raised) BMI due to high muscle mass. However, there must also be individual factors that determine why females with apparently similar body habitus and activity levels are discordant for HA. Indeed, HA females are more likely to harbor monoallelic gene variants associated with CHH than control populations, which may increase their susceptibility to environmental stressors (^1^). In clinical practice, functional HA is commonly linked to stress - whether metabolic, physical, or psychological - as well as weight loss resulting from reduced caloric intake or intensive physical activity (^61^).

It may be difficult to distinguish HA from CHH in women with primary amenorrhea, or from an organic pituitary process in secondary amenorrhea, but aside from the clinical history, there are some helpful indicators, including BMI (usually <23 kg/m^2^ in HA), congenital defects (present in 60-70% of CHH), fasted 9 AM anterior pituitary function (normal in CHH aside from LH and FSH; potentially abnormal in organic pituitary disease), potentially low free T3 and IGF1, but high-normal cortisol and GH in HA; iron studies (high ferritin and iron binding saturation in iron overload), MRI of the pituitary gland (normal in CHH and HA, but usually abnormal in CPHD and organic acquired hypopituitarism) and olfactory bulbs (absent or hypoplastic in KS), body composition by DXA or bioimpedance (low fat mass and/or high lean body mass in HA), and potentially also genomic evaluation as a final consideration.

##### Management

Removing or addressing the underlying cause of HA (where achievable) allows normal function of the HPG axis to resume with restoration of normal ovarian hormone secretion, menstruation, fertility and bone mass. Teaching has thus traditionally emphasized the primacy of behavioral (dietary, exercise or psychological) interventions (^62^-^64^). However, many women with HA find these adjustments to be unacceptable or unachievable, and others may already have experienced a significant impact on bone, sexual or urogenital health in the years prior to their initial consultation. Therefore, under these circumstances, the SfE recommends considering HRT at the initial consultation, which could either form a bridging therapy pending the outcome of behavioral interventions, or if there is no resolution, continued until the normal age of menopause. In any case, the recommendation for HRT should not be deferred for longer than 6-12 months beyond the initial discussion if there is no reso lution (^1^).

Similar principles apply to women with other forms of functional central FH, e.g. induced by opiates or hyperprolactinaemia. Although the initial aim would be to seek to achieve resolution of hyperprolactinaemia through dopamine-agonist treatment of prolactinoma or substitution of anti-dopaminergic or serotonergic drugs with alternative neuroleptics, or weaning of opiate medication, this strategy cannot be open-ended. Again, HRT should be recommended at the outset if there is little realistic possibility of achieving this aim (e.g., patient or psychiatrist unwilling to risk destabilizing mental health by substituting a prolactin-neutral drug; pain or addiction specialist unable to wean opiates), or if resumption of menstruation has not occurred within 6-12 months of the initial consultation.

Conventional ethinylestradiol-based combined oral contraceptive (COC) are best avoided due to the inferior bone protection they confer compared with transdermal estradiol, which is likely mediated by greater inhibition of liver IGF1 secretion, and greater thrombosis risk. Nevertheless, for sexually active women in whom pregnancy would not be desirable, it is vital to discuss contraception at the initial consultation, due to the potential for reproductive function to recover rapidly and unpredictably in all forms of functional CH. If other contraceptive options are not acceptable, then a 17β-estradiol-based COC (or if not available, systemic estradiol combined with progesterone only pill) could be prescribed as an alternative to HRT (^1^).

### Hormone treatment

#### Estrogen

Estrogen replacement therapy in women with FH is essential to maintain secondary sexual characteristics, optimize sexual functioning, and prevent vasomotor or neurocognitive symptoms, bone loss and fragility fractures, urogenital atrophy, and (although not proven) premature cardiovascular disease.

There are three primary types of estrogen formulations that exhibit different pharmacodynamics and activity at the α and β estrogen receptors: 17β-estradiol (= estradiol), ethinylestradiol (EE) found in most COCs, and conjugated equine estrogens (CEE). Only estradiol can be reliably measured by clinical assays, being the natural endogenous hormone. CEE contains a mixture of various xenoestrogenic compounds purified from pregnant mare urine, and EE is a synthetic analogue that has long been used in COCs. EE interacts with both α and β estrogen receptors, irreversibly inhibiting CYP enzymes involved in steroid metabolism and activating the renin-angiotensin system, resulting in a mechanism of action that diverges from normal physiology and that necessitates regular blood pressure monitoring (^65^). The COC is also inferior to HRT in respect of bone health in women with POI (^66^). Therefore, HRT based on 17β-estradiol (available in the form of tablets, patches, spray or gel) is now the preferred form, as per all current guidance (^1^-^3^), with data from both menopausal and transgender females indicating a lower rate of thrombosis, especially when administered transdermally (^1^). Hence, routine use of COC as a first-line form of HRT is no longer recommended unless there is a real risk of unwanted pregnancy and other forms of contraception are not acceptable (^1^-^3^). In this case, the SfE recommends one of the newer estradiol-based COCs that have the additional advantage of only a few blank days, rather than the traditional “pill-free week” during which zero estrogen is administered (^1^).

#### Progestins

Progesterone is required for endometrial protection in women with a uterus who have completed puberty, and a variety of progestins can be administered systemically in a sequential or continuous combined manner, or as an intrauterine system (IUS). Long-acting progestin contraceptive implants or depot injections could probably provide adequate endometrial protection as part of a continuous combined HRT regimen, but are unlicensed for this purpose. Progestins should be prescribed at the lowest dose that achieves endometrial protection, evidenced by the lack of unscheduled bleeding. This is because prolonged use of combined HRT in postmenopausal women confers a higher risk of breast cancer (greater with continuous combined than sequential), whereas that risk is much lower in hysterectomized women on estrogen monotherapy (^1^). Nevertheless, prolonged HRT use in women of premenopausal age with FH does not increase the risk of breast cancer above baseline for a eugonadal female (^22^,^67^). It has been plausibly suggested that the progestin dose should be adjusted in proportion to the estradiol dose, so that estradiol doses exceeding 2 mg orally or via gel, 100 mcg patches, or 3 sprays (4.59 mg) may require higher progestin doses than in standard preparations (^22^), but evidence is lacking (^1^).

All three guidelines (^1^-^3^) favor micronised progesterone (oral or vaginal; 100-200 mg daily for the first 12-14 days of each calendar month, or 100 mg daily taken continuously), or its stereoisomer dydrogesterone (oral; 10 mg daily for the first 12-14 days of each calendar month, or 5-10 mg daily taken continuously), as being metabolically neutral, improving sleep quality and potentially mitigating breast cancer risk due to pro-apoptotic and more antiproliferative effects on breast tissue (^68^-^70^). However, a levonorgestrel 52 mg IUS (approximately 21 mcg/day steady state release over 5 years) provides the best endometrial protection and, due to lesser systemic absorption, is likewise anticipated to have a lower associated breast cancer risk than older, more androgenic and procoagulant progestins, such as norethisterone acetate (NEA) and MPA (^1^). Moreover, for a female with disability, in whom it has not proven possible to avoid distressing cyclical or unscheduled bleeding, the insertion of a levonorgestrel IUS under general anesthetic can be transformative of quality of life. Nevertheless, despite the overall advantages of micronised progesterone or its stereoisomer dydrogesterone for most women, NEA in a continuous-combined regimen provides the next best progestin for endometrial protection after the levonorgestrel IUS and, when administered transdermally in the form of combined patches with estradiol, may also avoid the prothrombotic effect of oral NEA.

### Cautions and contraindications

A wide range of medical conditions are listed as contraindications to HRT (or COC in eugonadal women) (^71^), based on studies predominantly using oral CEE in postmenopausal women or EE-based COC in younger women. Therefore, multidisciplinary discussion may be necessary with other specialists (e.g., Medical and surgical Oncologists and Neurologists, Hematologists, Stroke Physicians and Cardiologists) and of course the patient herself. To this end, it is useful to first remind all parties that, unlike perimenopause, there is no long-term treatment alternative to HRT and no valid or reasonable non-treatment strategy for FH. Second, that modern transdermal estradiol plus “neutral” progestins have a negligible prothrombotic action compared with historic CEE plus MPA regimens, with good evidence for this both from older postmenopausal women and younger transgender females (^1^). Finally, it may be necessary to focus the minds of colleagues by posing the direct question: *“if this were a young women with regular periods, would your usual standard of care for treating [migraine with aura, stroke, venous thromboembolism (VTE), meningioma, hepatic adenoma, obesity, diabetes, major surgery or a strong family history of breast cancer, etc.] comprise precautionary medical or surgical oophorectomy or anti-estrogen treatment?”.* From this analysis, it becomes clearer that, for women with FH below the age for usual menopause, HRT is only contraindicated in two conditions: past or current breast cancer and active endometrial cancer.

For eugonadal women with BRCA1 or BRCA2 mutations, a risk-reducing bilateral salpingo-oophorectomy after completion of childbearing around age 45 years (the age threshold for early menopause) is recommended to significantly reduce the risk of ovarian, fallopian tube, breast and peritoneal cancers. Subject to patient choice, only the most severe BRCA1 mutations, causing complete loss or major disruption of protein structure of function, would prompt consideration of earlier oophorectomy (down to age 35 years), especially as prophylactic bilateral mastectomy more effectively reduces the risk of aggressive triple negative breast cancer (^72^). Therefore, younger women with FH who are BRCA carriers should not be routinely denied HRT, particularly as many will have already experienced years of untreated estrogen deficiency. It would be ideal to make such decisions after consulting with oncology colleagues.

Oncology guidance also suggests that vaginal estrogen is contraindicated in women with a history of breast cancer, but this is based entirely on precautionary principles and with no direct evidence of harm. Indeed, as systemic absorption is negligible (^73^) and no impact on breast cancer-specific mortality has been identified (^74^), the SfE supports the use of vaginal estrogen in FH, where breast cancer has been treated with curative intent and there are sexual or urogenital symptoms; especially in women taking tamoxifen (rather than aromatase inhibitors) or having ER-negative disease (^1^).

In the context of migraine with aura, stroke, angina, inherited procoagulant disorder, VTE (or strong family history thereof), obesity, type 2 diabetes, dyslipidemia, or cigarette smoking, oral estrogen and androgenic progestins are strongly discouraged, and transdermal estradiol with oral micronised progesterone, dydrogesterone or levonorgestrel IUS are instead recommended (^1^); if necessary, with the addition of long-term anticoagulation as per Hematologist advice. In relation to myocardial infarction, the SfE (^1^) suggests pausing HRT for 6 months if tolerated. This recommendation was based on the HERS study (^75^), albeit this was done with a prothrombotic HRT regimen based on CEE plus MPA.

### HRT treatment: doses, monitoring and timelines

There are no licensed HRT products specifically developed for younger women with FH. All standard HRT preparations, whether at low, intermediate or higher dose, were formulated as the lowest effective doses to achieve suppression of vasomotor symptoms in the majority of symptomatic postmenopausal women, and not to maintain long-term bone, muscle, sexual, urogenital, or cardiovascular health in younger women with FH over a period of up to four decades, and this is where registry data relating to transgender women can help fill gaps in our knowledge. All women of reproductive age experiencing hypogonadism for six months or more should have a baseline assessment of BMD and identification of any additional risk factors for low bone mineral density, with the findings informing the frequency of any future DXA scans (^1^).

HRT in postmenopausal women achieves effective fracture risk prevention even at standard doses, with the benefits comparable to those of bone-specific drugs, which cannot anyway be prescribed open-endedly due to diminishing benefit and greater side-effect profile beyond the authorized period. Therefore, except under exceptional circumstances (and subject to adequate calcium and vitamin D intake), HRT is considered the only recommended treatment for the prevention or treatment of osteoporosis and for fracture prevention in women with FH up to the normal age for menopause (^1^). Replacement can typically be continued to the normal menopausal age or beyond, depending on BMD and estrogen-deficiency symptoms and considering patient preference based on personalized counselling. Thereafter, bone management mirrors that in a woman who has reached the menopause at an expected age. However, women who experienced prolonged gaps in HRT are recommended to continue until the upper limit of normal age at menopause (5556 years), rather than the median age (51 years).

Some clinicians continue to recommend adjusting HRT doses based on patient symptoms or BMD and discourage monitoring serum estradiol concentrations, and the International POI guidance broadly supports this position, albeit suggesting that target serum estradiol level 200-400 pmol/L might be appropriate (^2^). In contrast, guidance for transgender women (^76^) mandates adjusting estradiol doses to achieve serum levels harmonized to the mid-follicular range for healthy young women (typically 300-600 pmol/L). The latest International Turner syndrome guidance (^3^) also recommends adjusting the final HRT dose to achieve serum levels very similar to those of the general population (350-550 pmol/L). The SfE’s guideline committee concluded that there was currently insufficient evidence to recommend whether routine testing of serum estradiol versus non-testing achieved better efficacy and safety outcomes in women with FH (^1^), but nevertheless identified circumstances in which measuring levels was likely to be beneficial, including the persistence of symptoms despite reasonable dose-adjustments, failure of BMD to improve on serial DXA scanning, or when adherence was doubtful, and identified either 350-550 or 300-600 pmol/L as being reasonable target ranges. However, compliance with treatment and correct use of medication should be ascertained prior to adjusting the dose of estradiol. Evaluation of endometrial thickness might be needed to evaluate vaginal bleeding, but should not be done on a routine basis (^1^). Adjusting the estradiol prescription to achieve these target ranges will require, however, doses that are 50-100% higher than the highest “standard” menopause HRT in around 50% of women (^77^).

Table 1 provides an overview of various 17β-estradiol preparations along with their equivalent dosages.

**Table 1:** Estrogen presentations and adverse effects

Intake	Presentation			Adverse effects
**Oral**	Tablets of 1 and 2 mg			Weight gain, nausea,
Approximate equivalence to transdermal Monitoring method	1.5 mg/day Bloods 4 hours post-dose			vomiting, breast tenderness, headache, increased risk of VTE and gallstones, higher TG, increased thyroid binding globulin.
**Transdermal**	Patch (mcg/day)	Gel sachet (mg/day) Gel pump (0.75 mg/ actuation)	Spray (1.53 mg/ actuation)	Neutral effect on blood lipids, thyroid and blood
Approximate equivalences Monitoring method	75 mcg/24 h Blood after 48 hours and prior to new patch	1 mg/day 2 actuations Bloods 4-6 hours after application and no gel on the arms	3 actuations Blood 2-8 hours post-application; avoid sample from application site	clots. May cause skin irritation.

Adverse symptoms related to HRT typically resolve in the first three months of treatment and include bleeding, headaches, breast tenderness, nausea, fatigue and mood changes. It should be noted that the use of HRT does not increase the risk of breast cancer before the age of natural menopause (^67^).

### HRT protocols for induction of puberty

Females presenting with absent or incomplete puberty require a highly specific therapeutic approach in order to achieve optimal breast and uterine development according to genetic potential, using incremental doses of estradiol monotherapy (rather than immediate full-dose estradiol + progestin hormone replacement - HRT) at the outset, and with the introduction of a progestin to confer long-term endometrial protection potentially delayed until both of these parameters have plateaued at a satisfactory level. In any case, longitudinal evaluation of uterine development through sonography provides reliable information on endometrial thickness as well as the uterine configuration, dimensions and volume. Starting these patients too early on full dose combined HRT (or COC) results in a smaller final uterine volume and final breast development that may never surpass Tanner B3 (^3^,^4^,^11^,^12^,^78^).

### Options for achieving parenthood

It is important to inform women that HRT does not serve as a method of contraception. While fertility is reduced in the vast majority of patients, it is crucial to discuss and prescribe contraceptive options for those wishing to avoid pregnancy and, if other options such as levonorgestrel IUS are not acceptable, an estradiolbased COC could be used as an alternative to conventional HRT.

Fertility restoration is significantly influenced by the underlying cause of hypogonadism. In cases of HA, a comprehensive approach that includes proper nutrition, stress management, physical activity in moderation, and psychological intervention, such as cognitive behavioral therapy, is often deemed most effective. While implementing lifestyle changes can be challenging, they are often the ideal course of action and may successfully restore fertility in these patients. If lifestyle modifications are not achieved and a hypogonadal state persists for over 6 to 12 months, a discussion regarding HRT should be made. Although leptin has shown promise in restoring ovulation in certain women affected by HA (^59^) natural cycle monofollicular gonadotropin ovulation induction (GnOI) or pulsatile GnRH if available, is generally considered the primary treatment approach when behavioral strategies are unsuccessful (^79^), and is of course first line for women with organic central FH. Other things being equal, the cumulative likelihood of achieving pregnancy over the course of 3-6 cycles of GnRH parallels that of normally fertile couples, after which assisted reproduction techniques (ART) can be deployed. Crucially, a low AMH or antral follicle count in a woman with central FH should not be considered pathognomonic of a low ovarian reserve (rather as expected for unstimulated granulosa cells) and should not deter GnOI or ART. However, for women with panhypopituitarism and especially with vasopressin deficiency (a surrogate marker for oxytocin deficiency), the risk of pregnancy and labor are high, and thus specialist MDT oversight is required throughout pregnancy (^80^), much as for women with TS.

In TS (and other forms of POI), oocyte donation is usually necessary to achieve pregnancy. However, additional factors that may influence fertility or pregnancy outcome should always be investigated before proceeding to treatment, such as male sperm, tubal and uterine integrity and other external factors. In TS, no treatment should be offered until a comprehensive cardiovascular assessment has been undertaken, due to the heightened risk of aortic dissection in pregnancy (^3^).

## CLOSING REMARKS

**Figure 1:**
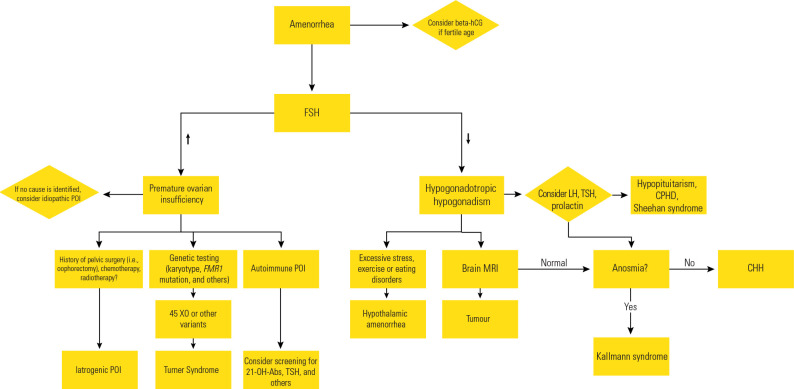
Suggested flowchart for investigation and management.

In conclusion, a serum estradiol concentration persistently < 200 pmol/L and/or FSH > 25 IU/L in the presence of amenorrhea and relevant clinical features is consistent with FH, even when another potential cause for amenorrhea is present or has been proposed. HRT is recommended for all women with FH of premenopausal age, including those with a reversible cause, if they are experiencing sustained amenorrhea. Ascertaining the likely etiology of FH, especially distinguishing between central FH and POI, is critical to ensure appropriate investigation and management, including accurate exposition of fertility options. HRT based on native 17β-estradiol should be prescribed in preference to synthetic or equine estrogens and, in women with a uterus, in combination with micronised progesterone, dydrogesterone or levonorgestrel IUS. HRT aims to provide physiological estrogen replacement to young women with FH, and so significantly higher doses of estradiol may be required than are contained in standard preparations formulated for natural postmenopause, and with no arbitrary limits placed upon the duration of HRT up to the normal age range for menopause (**Figure 1**).

There is no evidence that HRT increases risk of breast cancer (above being female) in young women with FH. Neither thrombotic risk factors (e.g., obesity, smoking, migraine aura) or events (stroke, angina, myocardial infarction), family history, nor genetic tumor predisposition (e.g., BRCA 1 and 2 mutations) should be reasons to deny HRT to a younger woman with FH, although HRT should ideally be paused for 6 months following MI. However, in these circumstances, transdermal routes of estradiol administration should be used first-line.

Guidelines are discordant as to whether serum estradiol levels should (International Turner) or should not (International POI) be measured routinely as part of routine treatment monitoring, with the Society for Endocrinology unable to make a firm recommendation either way based on current evidence. However, if testing is done, then an estradiol concentration in the 350-550 pmol/L range as per Turner’s guidance would appear reasonable. Levonorgestrel IUS or 17β-estradiol-based contraceptives are ideal for women with reversible FH wanting contraception. Women with FH should have an assessment of BMD and identification of any additional risk factors for low bone mineral density. GnOI is recommended as the first line fertility therapy for women with central forms of FH.

## Data Availability

datasets related to this article will be available upon request to the corresponding author.

## References

[1] Jayasena CN, Devine K, Barber K, Comninos AN, Conway GS, Crown A (2024). Society for endocrinology guideline for understanding, diagnosing
and treating female hypogonadism. Clin Endocrinol (Oxf).

[2] Panay N, Anderson RA, Bennie A, Cedars M, Davies M, Ee C, ESHRE, ASRM, CREWHIRL and IMS Guideline Group on POI (2024). Evidencebased guideline: premature ovarian
insufficiency. Climacteric.

[3] Gravholt CH, Andersen NH, Christin-Maitre S, Davis SM, Duijnhouwer A, Gawlik A (2024). Clinical practice guidelines for the care of girls and women with
Turner syndrome. Eur J Endocrinol.

[4] Howard SR, Quinton R. (2023). Outcomes and experiences of adults with congenital hypogonadism
can inform improvements in the management of delayed puberty. J Pediatr Endocrinol Metab.

[5] Deeks AA, Gibson-Helm M, Teede H, Vincent A. (2011). Premature menopause: A comprehensive understanding of
psychosocial aspects. Climacteric.

[6] Minis E, Pinero L, Bhatt S, O’Besso V, Douglas NC, Morelli SS. (2022). Primary Ovarian Insufficiency: Time to Diagnosis and a Review of
Current Literature. Clin Exp Obstet Gynecol.

[7] Zhu D, Chung HF, Dobson AJ, Pandeya N, Giles GG, Bruinsma F (2019). Age at natural menopause and risk of incident cardiovascular
disease: a pooled analysis of individual patient data. Lancet Public Health.

[8] Liao H, Cheng J, Pan D, Deng Z, Liu Y, Jiang J (2023). Association of earlier age at menopause with risk of incident
dementia, brain structural indices and the potential mediators: a
prospective community-based cohort study. EClinicalMedicine.

[9] Sochocka M, Karska J, Pszczołowska M, Ochnik M, Fułek M, Fułek K (2023). Cognitive Decline in Early and Premature
Menopause. Int J Mol Sci.

[10] Anagnostis P, Lambrinoudaki I, Goulis DG. (2025). Is Early Menopause a Different Entity from Premature Ovarian
Insufficiency?. Clin Endocrinol (Oxf).

[11] Macias H, Hinck L. (2012). Mammary gland development. Wiley Interdiscip Rev Dev Biol.

[12] Romo RC, Quinton R, Owen K, Peacock A, Boal R, Wood C (2025). Optimal Uterine Growth During Pubertal Induction in Hypogonadal
Females Is Dependent on Type and Duration of Unopposed Oestrogen
Treatment. Clin Endocrinol (Oxf).

[13] Federici S, Goggi G, Quinton R, Giovanelli L, Persani L, Cangiano B (2022). New and Consolidated Therapeutic Options for Pubertal Induction
in Hypogonadism: In-depth Review of the Literature. Endocr Rev.

[14] Skorupskaite K, George JT, Anderson RA. (2014). The kisspeptin-GnRH pathway in human reproductive health and
disease. Hum Reprod Update.

[15] Cangiano B, Swee DS, Quinton R, Bonomi M. (2021). Genetics of congenital hypogonadotropic hypogonadism:
peculiarities and phenotype of an oligogenic disease. Hum Genet.

[16] Critchley HOD, Maybin JA, Armstrong GM, Williams ARW. (2020). Physiology of the endometrium and regulation of
menstruation. Physiol Rev.

[17] Mills EG, Yang L, Nielsen MF, Kassem M, Dhillo WS, Comninos AN. (2021). The Relationship between Bone and Reproductive Hormones beyond
Estrogens and Androgens. Endocr Rev.

[18] Xiang D, Liu Y, Zhou S, Zhou E, Wang Y. (2021). Protective Effects of Estrogen on Cardiovascular Disease Mediated
by Oxidative Stress. Oxid Med Cell Longev.

[19] Nie G, Yang X, Wang Y, Liang W, Li X, Luo Q (2022). The Effects of Menopause Hormone Therapy on Lipid Profile in
Postmenopausal Women: A Systematic Review and Meta-Analysis. Front Pharmacol.

[20] Ali N, Sohail R, Jaffer SR, Siddique S, Kaya B, Atowoju I (2023). The Role of Estrogen Therapy as a Protective Factor for
Alzheimer’s Disease and Dementia in Postmenopausal Women: A Comprehensive
Review of the Literature. Cureus.

[21] Ruediger SL, Koep JL, Keating SE, Pizzey FK, Coombes JS, Bailey TG. (2021). Effect of menopause on cerebral artery blood flow velocity and
cerebrovascular reactivity: Systematic review and
meta-analysis. Maturitas.

[22] Schulman IH, Raij L. (2006). Salt sensitivity and hypertension after menopause: Role of nitric
oxide and angiotensin II. Am J Nephrol.

[23] Mauvais-Jarvis F, Clegg DJ, Hevener AL. (2013). The Role of Estrogens in Control of Energy Balance and Glucose
Homeostasis. Endocr Rev.

[24] Otsuka N, Tong ZB, Vanevski K, Tu W, Cheng MH, Nelson LM. (2011). Autoimmune Oophoritis with Multiple Molecular Targets Mitigated
by Transgenic Expression of Mater. Endocrinology.

[25] Fukami M. (2023). Ovarian dysfunction in women with Turner syndrome. Front Endocrinol (Lausanne).

[26] Blackwood JD, Martin MA, Jafri D, Rehmann D. (2025). Freezing Time and Preserving Hope: Ovarian Tissue
Cryopreservation for Girls with Turner Syndrome. Georgetown Medical Review.

[27] Szeliga A, Calik-Ksepka A, Maciejewska-Jeske M, Grymowicz M, Smolarczyk K, Kostrzak A (2021). Autoimmune diseases in patients with premature ovarian
insufficiency-our current state of knowledge. Int J Mol Sci.

[28] Bidet M, Bachelot A, Bissauge E, Golmard JL, Gricourt S, Dulon J (2011). Resumption of ovarian function and pregnancies in 358 patients
with premature ovarian failure. J Clin Endocrinol Metab.

[29] Blumenfeld Z, Mischari O, Schultz N, Boulman N, Balbir-Gurman A. (2011). Gonadotropin Releasing Hormone Agonists May Minimize
Cyclophosphamide Associated Gonadotoxicity in SLE and Autoimmune
Diseases. Semin Arthritis Rheum.

[30] Chow EJ, Stratton KL, Leisenring WM, Oeffinger KC, Sklar CA, Donaldson SS (2016). Pregnancy after chemotherapy in male and female survivors of
childhood cancer treated between 1970 and 1999: a report from the Childhood
Cancer Survivor Study cohort. Lancet Oncol.

[31] Spath MA, Braat DDM. (2019). Iatrogenic and non-iatrogenic causes of female fertility loss
that may indicate fertility preservation. Acta Obstet Gynecol Scand.

[32] Himpe J, Lammerant S, Van den Bergh L, Lapeire L, De Roo C. (2023). The Impact of Systemic Oncological Treatments on the Fertility of
Adolescents and Young Adults-A Systematic Review. Life (Basel).

[33] Nelson SM, Davis SR, Kalantaridou S, Lumsden MA, Panay N, Anderson RA. (2023). Anti-Müllerian hormone for the diagnosis and prediction of
menopause: a systematic review. Hum Reprod Update.

[34] Anderson RA, Cameron D, Clatot F, Demeestere I, Lambertini M, Nelson SM (2022). Anti-Müllerian hormone as a marker of ovarian reserve and
premature ovarian insufficiency in children and women with cancer: a
systematic review. Hum Reprod Update.

[35] Cameron DA, Anderson R, Clatot F, Demeestere I, Lambertini M, Nelson SM (2021). Anti-Müllerian hormone (AMH) as a marker of ovarian
reserve and premature ovarian insufficiency (POI) in children and women with
cancer: A systematic review. J Clin Oncol.

[36] Rozen G, Rogers P, Chander S, Anderson R, McNally O, Umstad M (2020). Clinical summary guide: Reproduction in women with previous
abdominopelvic radiotherapy or total body irradiation. Hum Reprod Open.

[37] Heddar A, Ogur C, Da Costa S, Braham I, Billaud-Rist L, Findlinki N (2022). Genetic landscape of a large cohort of Primary Ovarian
Insufficiency: New genes and pathways and implications for personalized
medicine. EbioMedicine.

[38] Leehey MA. (2009). Fragile X-associated tremor/ataxia syndrome: Clinical phenotype,
diagnosis, and treatment. J Investig Med.

[39] Ke H, Tang S, Guo T, Hou D, Jiao X, Li S (2023). Landscape of pathogenic mutations in premature ovarian
insufficiency. Nat Med.

[40] Shekari S, Stankovic S, Gardner EJ, Hawkes G, Kentistou KA, Beaumont RN (2023). Penetrance of pathogenic genetic variants associated with
premature ovarian insufficiency. Nat Med.

[41] Evangelinakis N, Geladari EV, Geladari CV, Kontogeorgi A, Papaioannou GK, Peppa M (2024). The influence of environmental factors on premature ovarian
insufficiency and ovarian aging. Maturitas.

[42] Boehm U, Bouloux PM, Dattani MT, De Roux N, Dodé C, Dunkel L (2015). Expert consensus document: European Consensus Statement on
congenital hypogonadotropic hypogonadism - pathogenesis, diagnosis and
treatment. Nat Rev Endocrinol.

[43] Caronia LM, Martin C, Welt CK, Sykiotis GP, Quinton R, Thambundit A (2011). A Genetic Basis for Functional Hypothalamic
Amenorrhea. N Engl J Med.

[44] Delaney A, Burkholder AB, Lavender CA, Plummer L, Mericq V, Merino PM (2021). Increased Burden of Rare Sequence Variants in GnRHAssociated
Genes in Women with Hypothalamic Amenorrhea. J Clin Endocrinol Metab.

[45] Balasubramanian R, Choi JH, Francescatto L, Willer J, Horton ER, Asimacopoulos EP (2014). Functionally compromised CHD7 alleles in patients with isolated
GnRH deficiency. Proc Natl Acad Sci U S A.

[46] Raivio T, Avbelj M, McCabe MJ, Romero CJ, Dwyer AA, Tommiska J (2012). Genetic overlap in Kallmann syndrome, combined pituitary hormone
deficiency, and septo-optic dysplasia. J Clin Endocrinol Metab.

[47] Dzemaili S, Tiemensma J, Quinton R, Pitteloud N, Morin D, Dwyer AA. (2017). Beyond hormone replacement: Quality of life in women with
congenital hypogonadotropic hypogonadism. Endocr Connect.

[48] Young J, Xu C, Papadakis GE, Acierno JS, Maione L, Hietamäki J (2019). Clinical Management of Congenital Hypogonadotropic
Hypogonadism. Endocr Rev.

[49] Quinton R, Duke VM, Robertson A, Kirk JMW, Matfin G, De Zoysa PA (2001). Idiopathic gonadotrophin deficiency: Genetic questions addressed
through phenotypic characterization. Clin Endocrinol (Oxf).

[50] Fleseriu M, Christ-Crain M, Langlois F, Gadelha M, Melmed S. (2024). Hypopituitarism. Lancet.

[51] Fang Q, George AS, Brinkmeier ML, Mortensen AH, Gergics P, Cheung LYM (2016). Genetics of combined pituitary hormone deficiency: Roadmap into
the genome era. Endocr Rev.

[52] Castinetti F, Reynaud R, Saveanu A, Jullien N, Quentien MH, Rochette C (2016). Mechanisms in Endocrinology: An update in the genetic aetiologies
of combined pituitary hormone deficiency. Eur J Endocrinol.

[53] Bando H, Urai S, Kanie K, Sasaki Y, Yamamoto M, Fukuoka H (2022). Novel genes and variants associated with congenital pituitary
hormone deficiency in the era of next-generation sequencing. Front Endocrinol (Lausanne).

[54] Ryabets-Lienhard A, Stewart C, Borchert M, Geffner ME. (2016). The Optic Nerve Hypoplasia Spectrum: Review of the Literature and
Clinical Guidelines. Adv Pediatr.

[55] van Ravenswaaij-Arts C, Martin DM. (2017). New insights and advances in CHARGE syndrome: Diagnosis,
etiologies, treatments, and research discoveries. Am J Med Genet C Semin Med Genet.

[56] Balasubramanian R, Crowley WF. (2017). Reproductive endocrine phenotypes relating to CHD7 mutations in
humans. Am J Med Genet C Semin Med Genet.

[57] Tauber M, Hoybye C. (2021). Endocrine disorders in Prader-Willi syndrome: a model to
understand and treat hypothalamic dysfunction. Lancet Diabetes Endocrinol.

[58] Patel AH, Koysombat K, Pierret A, Young M, Comninos AN, Dhillo WS (2024). Kisspeptin in functional hypothalamic amenorrhea: Pathophysiology
and therapeutic potential. Ann N Y Acad Sci.

[59] Welt CK, Chan JL, Bullen J, Murphy R, Smith P, DePaoli AM (2004). Recombinant Human Leptin in Women with Hypothalamic
Amenorrhea. N Engl J Med.

[60] Jayasena CN, Abbara A, Veldhuis JD, Comninos AN, Ratnasabapathy R, De Silva A (2014). Increasing LH pulsatility in women with hypothalamic amenorrhoea
using intravenous infusion of kisspeptin-54. J Clin Endocrinol Metab.

[61] Speroff L, Glass R, Kase N. (1999). Clinical gynecologic endocrinology and infertility.

[62] Pedreira CC, Maya J, Misra M. (2022). Functional hypothalamic amenorrhea: Impact on bone and
neuropsychiatric outcomes. Front Endocrinol (Lausanne).

[63] Mountjoy M, Sundgot-Borgen J, Burke L, Ackerman KE, Blauwet C, Constantini N (2018). International Olympic Committee (IOC) Consensus statement on
relative energy deficiency in sport (red-s): 2018 update. Int J Sport Nutr Exerc Metab.

[64] Gordon CM, Ackerman KE, Berga SL, Kaplan JR, Mastorakos G, Misra M (2017). Functional hypothalamic amenorrhea: An endocrine society clinical
practice guideline. J Clin Endocrinol Metab.

[65] Swee DS, Javaid U, Quinton R. (2019). Estrogen Replacement in Young Hypogonadal Women-Transferrable
Lessons from the Literature Related to the Care of Young Women with
Premature Ovarian Failure and Transgender Women. Front Endocrinol (Lausanne).

[66] Cartwright B, Robinson J, Seed PT, Fogelman I, Rymer J. (2016). Hormone replacement therapy versus the combined oral
contraceptive pill in premature ovarian failure: A randomized controlled
trial of the effects on bone mineral density. J Clin Endocrinol Metab.

[67] Lambrinoudaki I. (2020). Menopausal hormone therapy and breast cancer risk: All
progestogens are not the same. Case Rep Womens Health.

[68] Stahlberg C, Pedersen AT, Lynge E, Andersen ZJ, Keiding N, Hundrup YA (2004). Increased risk of breast cancer following different regimens of
hormone replacement therapy frequently used in Europe. Int J Cancer.

[69] Seeger H, Mueck AO. (2008). Are the progestins responsible for breast cancer risk during
hormone therapy in the postmenopause? Experimental vs. clinical
data. J Steroid Biochem Mol Biol.

[70] Webber L, Davies M, Anderson R, Bartlett J, Braat D, Cartwright B, European Society for Human Reproduction and Embryology (ESHRE)
Guideline Group on POI (2016). ESHRE Guideline: Management of women with premature ovarian
insufficiency. Hum Reprod.

[71] Bennett S, Mathur R. (2024). Hormone Replacement Therapy. Obstet Gynaecol Reprod Med.

[72] Petrucelli N, Daly MB, Pal T. (2025). BRCA1- and BRCA2-Associated Hereditary Breast and Ovarian
Cancer. GeneReviews®.

[73] Santen RJ, Mirkin S, Bernick B, Constantine GD. (2020). Systemic estradiol levels with low-dose vaginal
estrogens. Menopause.

[74] McVicker L, Labeit AM, Coupland CAC, Hicks B, Hughes C, McMenamin Ú (2024). Vaginal Estrogen Therapy Use and Survival in Females with Breast
Cancer. JAMA Oncol.

[75] Hulley S, Grady D, Bush T, Furberg C, Herrington D, Riggs B (1998). Randomized trial of estrogen plus progestin for secondary
prevention of coronary heart disease in postmenopausal women. Heart and
Estrogen/progestin Replacement Study (HERS) Research Group. JAMA.

[76] Seal LJ. (2016). A review of the physical and metabolic effects of cross-sex
hormonal therapy in the treatment of gender dysphoria. Ann Clin Biochem.

[77] Howarth S, Quinton R, Mohammed A. (2023). Estradiol treatment in a large cohort of younger women with
congenital hypogonadism: how much is enough?. Clin Endocrinol (Oxf).

[78] Federici S, Goggi G, Quinton R, Giovanelli L, Persani L, Cangiano B (2022). New and Consolidated Therapeutic Options for Pubertal Induction
in Hypogonadism: In-depth Review of the Literature. Endocr Rev.

[79] White DM, Hardy K, Lovelock S, Franks S. (2018). Low-dose gonadotropin induction of ovulation in anovulatory
women: Still needed in the age of IVF. Reproduction.

[80] Overton CE, Davis CJ, West C, Davies MC, Conway GS. (2002). High risk pregnancies in hypopituitary women. Hum Reprod.

